# Liposomal bupivacaine administration is not superior to traditional periarticular injection for postoperative pain management following total knee arthroplasty: a meta-analysis of randomized controlled trials

**DOI:** 10.1186/s13018-023-03699-4

**Published:** 2023-03-16

**Authors:** Jian-Jiun Chen, Yun-Che Wu, Jun-Sing Wang, Cheng-Hung Lee

**Affiliations:** 1 Department of Orthopedics, Taipei Veterans General Hospital, Taipei, Taiwan; 2 Department of Orthopedics, Taichung Veterans General Hospital, No.1650, Sec. 4, Taiwan Boulevard, Taichung, 40705 Taiwan; 3 Division of Endocrinology and Metabolism, Department of Internal Medicine, Taichung Veterans General Hospital, No.1650, Sec. 4, Taiwan Boulevard, Taichung, 40705 Taiwan; 4 Ph.D. Program in Translational Medicine, National Chung Hsing University, Taichung, Taiwan; 5 Department of Post-Baccalaureate Medicine, College of Medicine, National Chung Hsing University, Taichung, Taiwan; 6 Department of Food Science and Technology, Hung Kuang University, Taichung, Taiwan

**Keywords:** Total knee arthroplasty, Liposomal bupivacaine, Traditional periarticular injection

## Abstract

**Background:**

Liposomal bupivacaine (LB) is a relatively new formulation that slowly releases bupivacaine to extend its efficacy for 72–96 h. It is inconclusive whether LB offers better efficacy than traditional periarticular injection (TPAI) following total knee arthroplasty (TKA).

**Methods:**

Relevant randomized controlled trials (RCTs) were searched using electronic databases, including PubMed, Cochrane Library, EMBASE, and Web of Science. Review Manager 5.4.1 was used for calculations.

**Results:**

Sixteen RCTs were included in this meta-analysis. LB had better effects on morphine consumption equivalents during postoperative 24–48 h than TPAI. No significant difference was observed in pain relief, incidence of nausea and vomiting, or length of hospital stay between the two groups.

**Conclusion:**

LB administration during TKA is not superior to TPAI. Studies with larger sample size are needed to validate our findings.

*PROSPERO registration number*: CRD42022355094.

## Introduction

Total knee arthroplasty (TKA) is the most successful and common orthopedic surgery for decreasing pain in patients with osteoarthritis. The number of TKA conducted each year in the USA has been more than 700,000 [1]. Postoperative pain often has negative impact on patient outcomes. Patients with persistent pain cannot undergo early rehabilitation. This results in a decrease in knee joint function, slow recovery time, and delayed hospital discharge [2]. Thus, coping with postoperative pain is critical, but there is currently no gold standard treatment [3].

Kerr and Kohan first described periarticular multimodal drug injection (PMDI), a technique that combines multiple analgesics or anesthetic agents for injection into periarticular spaces during surgery [4–6]. The technique is simple and effective. PMDI can effectively reduce postoperative pain, decrease the demand for systemic analgesics, and consequently decrease the side effects of systemic analgesics [7–12].

Which treatment is the most effective analgesic or anesthetic remains inconclusive. Liposomal bupivacaine (LB; Exparel; Pacira Pharmaceuticals, Parsippany, NJ, USA), a formulation composed of multivesicular liposomes that contain bupivacaine, was approved by the FDA in 2011. The formulation allows bupivacaine to be released more slowly, extending its efficacy to 72–96 h [13]. LB is administered intraoperatively into the surgical wound. This medicine has already been applied safely in several procedures, including TKA and augmentation mammoplasty [14]. LB has been claimed to achieve a better effect on postoperative pain control, lower analgesic rescue dose, lower opioid-related adverse effects (ORAE), and a shorter length of hospital stay than the traditional bupivacaine injection [15]. To analyze the efficacy of LB for TKA and compare it with that of standard agents, the best approach is to use a periarticular injection approach to administer both LB and standard agents following TKA; the advantages are the same administration method and minimal confounding factors [16].

Uncertainty regarding the differences in outcomes between LB and traditional periarticular injection (TPAI) following TKA has been demonstrated in several studies. Only two meta-analyses focused on randomized controlled trials (RCTs) were published in 2019, and there are some data gaps [17, 18]. The conclusion of Yayac et al. [17] revealed that LB offers no advantages over other analgesics, and Liu et al. [18] found no significant difference on the visual analog scale (VAS) after comparing LB to TPAI; however, LB was associated with lower opioid consumption and incidence of nausea and vomiting.

The value of spending more than 10 times the money to use LB rather than TPAI for as-yet-unproven benefits is dubious [19]. As a result, the present study aimed to analyze more valid RCTs, including those published before May 2022, and to fill gaps in the data of previous studies.

## Patient and method

This study was conducted per The Preferred Reporting Items for Systematic Reviews and Meta-Analyses (PRISMA) guidelines [20].

### Search strategy

PubMed, the Cochrane Library, EMBASE, and Web of Science were searched up to May 2022. The search terms included “total knee arthroplasty OR replacement” AND “liposomal bupivacaine” AND “local infiltration OR periarticular injection OR periarticular infiltration.” Furthermore, the reference lists of included studies were searched to identify potentially eligible studies. All the searches were conducted independently by two authors, and disagreements were resolved by the third author.

### Study selection

Only “Randomized Controlled Trials” comparing an LB group with a control group were identified. Studies were considered eligible only if they met these criteria: (1) Patient underwent primary TKA. (2) Intervention group received a periarticular LB injection. (3) Control group received TPAI, including standard bupivacaine and cocktail (ropivacaine, morphine, ketorolac, epinephrine, etc.) (4) At least one of the following outcomes was reported: postoperative pain score with rest or activity, opioid consumption, ORAE (nausea and vomiting), and length of hospital stay.

All potentially eligible studies and relevant citations were screened by two authors independently for inclusion. Disagreements were resolved by the third author.

### Data abstraction and quality assessment

A standard form was designed by two authors to screen the relevant data in each included study. A Microsoft Excel database was used for data collection. The following data were extracted: (1) patient characteristics (age, sex, and other baseline characteristics), (2) interventions (LB, bupivacaine, or cocktail), (3) outcomes (primary outcome: the VAS score; secondary outcomes: opioid consumption in oral morphine equivalents, the incidence of nausea and vomiting, and the length of hospital stay). Missing data were obtained by contacting corresponding authors. If variability data could not be obtained by studies or authors, the Cochrane Handbook for Systematic Reviews of Interventions was followed to calculate standard deviations by using *p* values and confidence intervals [21].

Two authors independently assessed the risk of bias associated with the following factors: random sequence generation, allocation concealment, blinding, incomplete outcome data, selective reporting, and other potential sources of bias. Additionally, all authors’ conflict of interest statements were assessed. Disagreements were determined by the third author.

The quality of evidence for each outcome was evaluated using the Grading of Recommendations Assessment, Development and Evaluation (GRADE) criteria [22].

### Data analysis

Data were analyzed using the latest version of Review Manager (5.4.1, released in September 2020). The mean differences (MDs) were used to assess the effects of treatment for continuous outcomes. The risk ratios (RRs) were used to weigh the effect size of dichotomous outcomes. The standardized mean difference (SMD) was used to evaluate the morphine consumption equivalents due to the high degree of variability. Significant heterogeneity was considered when *p* ≤ 0.1 or *I*^2^ > 50%. A fixed-effects model was used for the study groups without significant heterogeneity. A random-effects model was used to ensure the robustness of the model.

In the part of postoperative VAS score, the control group was classified into standard bupivacaine and cocktail groups, and subgroup analyses were performed to eliminate any possible risk of bias.

## Results

### Search results

**Fig. 1:**
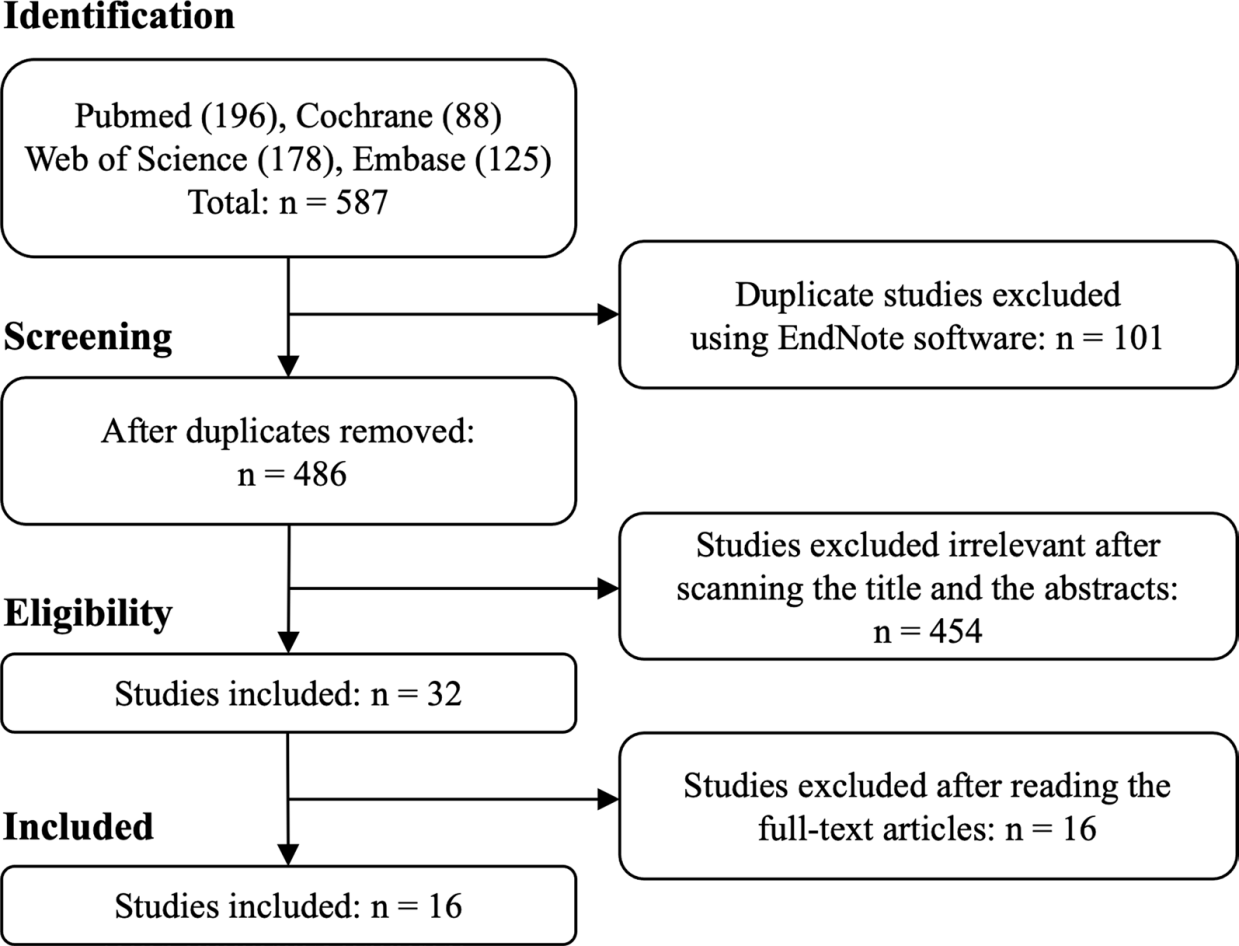
Flowchart of literature selection procedure

A total of 587 citations were identified. Using EndNote software, 101 duplicate citations were excluded. After scanning the titles and abstracts, 454 citations were excluded, and 16 citations were eliminated after reading the full texts. Finally, 16 RCTs met the inclusion criteria of this study (Fig. 1) [15, 19, 23–36].

### Characteristics of included studies

A total of 16 RCTs with 1629 participants were involved. Ten RCTs used standard bupivacaine as the control drug [15, 23, 24, 26, 29, 31, 33–36], whereas 6 RCTs used a cocktail. All the RCTs were conducted in the USA. Six RCTs did not perform power analysis to determine the optimal sample size [25, 28, 31, 34–36]. The follow-up period ranged from 24 h to 8 weeks (Table 1).

**Table 1:** Characteristics of included studies

Study (year)	Setting	LB/TPAI					AN	Treatment group	Control group	Follow-up	PA
		No.	Age	Male	Female	BMI					
Bramlett 2012 [23]	USA	25/34	61.1/62.2	13/11	12/23	31.2/31.5	GA	LB 266 mg	Bup 150 mg	36 days	Y
Schroer 2015 [24]	USA	58/53	67/68.6	24/21	34/32	32/32	SA/GA	LB 266 mg, Bup 75 mg	Bup 150 mg	3 weeks	Y
Collis 2016 [25]	USA	54/51	63.7/63.5	25/14	29/37	34.1/35.7	GA	LB 266 mg	Rop 246.25 mg, Epi 0.5 mg, Ket 30 mg, Clo 0.08 mg	8 weeks	N
Jain 2016 [26]	USA	63/62	68.3/67.5	19/17	44/45	33.3/33.3	SA	LB 266 mg	Bup 75 mg, Epi 0.15 mg, Mor 10 mg	NM	Y
Schwarzkopf 2016 [27]	USA	20/18	63/59	7/10	13/8	29.3/29.5	SA	LB 266 mg, Bup 50 mg	Rop 246.25 mg, Epi 0.5 mg, Clo 80 mg, Tor 30 mg	NM	Y
Snyder 2016 [28]	USA	35/35	67.3/65.6	22/15	13/20	30.68/31.29	SA/GA	LB 266 mg	Rop 400 mg, Epi 0.6 mg, Ket 30 mg, Mor 5 mg	10 days	N
Alijanipour 2017 [29]	USA	59/59	64.3/64.9	29/27	30/32	32.3/28.7	SA	LB 266 mg, Epi 0.5 mg	Bup 50 mg, Epi 0.1 mg	6 weeks	Y
Declaire 2017 [30]	USA	47/49	69.7/67.7	21/21	26/28	31.5/31.9	SA/GA	LB 266 mg, Bup, Epi, Ket, Mor	Rop, Epi, Ket, Mor	NM	Y
Mont 2017 [15]	USA	70/69	66/66	27/30	43/39	32.4/31.3	SA	LB 266 mg, Bup 100 mg	Bup 100 mg	NM	Y
Smith 2017 [31]	USA	104/96	66/66	54/28	50/68	31.5/31.6	SA	LB 266 mg	Bup	6 weeks	N
Danoff 2018 [32]	USA	29/29	62.9/62.9	15/15	14/14	30.4/30.4	SA	LB 266 mg, Bup 75 mg	Rop 250 mg, Epi 0.5 mg, Ket 30 mg, Clo 0.08 mg	6 weeks	Y
Schumer 2018 [36]	USA	66/64	NM	NM	NM	NM	SA	LB 266 mg, Bup 1 mg/kg	Bup 150 mg	6 weeks	N
Suarez 2018 [33]	USA	52/52	68.1/67.3	19/26	33/26	30.8/32.01	SA	LB 266 mg, Bup 75 mg	Rop 246.25 mg, Epi 0.5 mg, Ket 30 mg, Clo 0.08 mg	6 weeks	Y
Zlotnicki 2018 [34]	USA	38/40	63.2/64.3	19/14	19/26	35.5/35.4	SA/GA	LB 266 mg	Bup 100 mg	NM	N
Dysart 2019 [35]	USA	70/69	66/66	29/28	41/41	NM	SA	LB 266 mg, Bup 100 mg	Bup 100 mg	24 h	N
Hyland 2019 [19]	USA	30/29	65.0/61.2	14/13	16/16	NM	GA + ACB	LB 266 mg	Rop 40 mg, Ket 30 mg, Mor 10 mg, Methy 40 mg	NM	Y

G1, treatment group; G2, control group; AN, anesthesia; SA, spinal anesthesia, GA, general anesthesia; ACB, adductor canal nerve block; PA, power analysis; Bup, bupivacaine; Rop, ropivacaine; Epi, epinephrine; Ket, ketorolac; Mor, morphine; Methy, methylprednisolone acetate; Clo, clonidine, Tor, toradol

### Study quality

Regarding randomization, three studies used random number tables [28, 31, 33], two studies used Microsoft Excel software [29, 30], four studies used centralized randomization systems [15, 19, 23, 35], and the randomization method was not mentioned in the other studies. Only two studies reported the concealment of allocation [19, 29]. Most studies were blind to participants and outcome assessors but not the surgeons who injected the drugs. Only six studies were blind to the surgeons [15, 23, 30–32, 35].

**Fig. 2:**
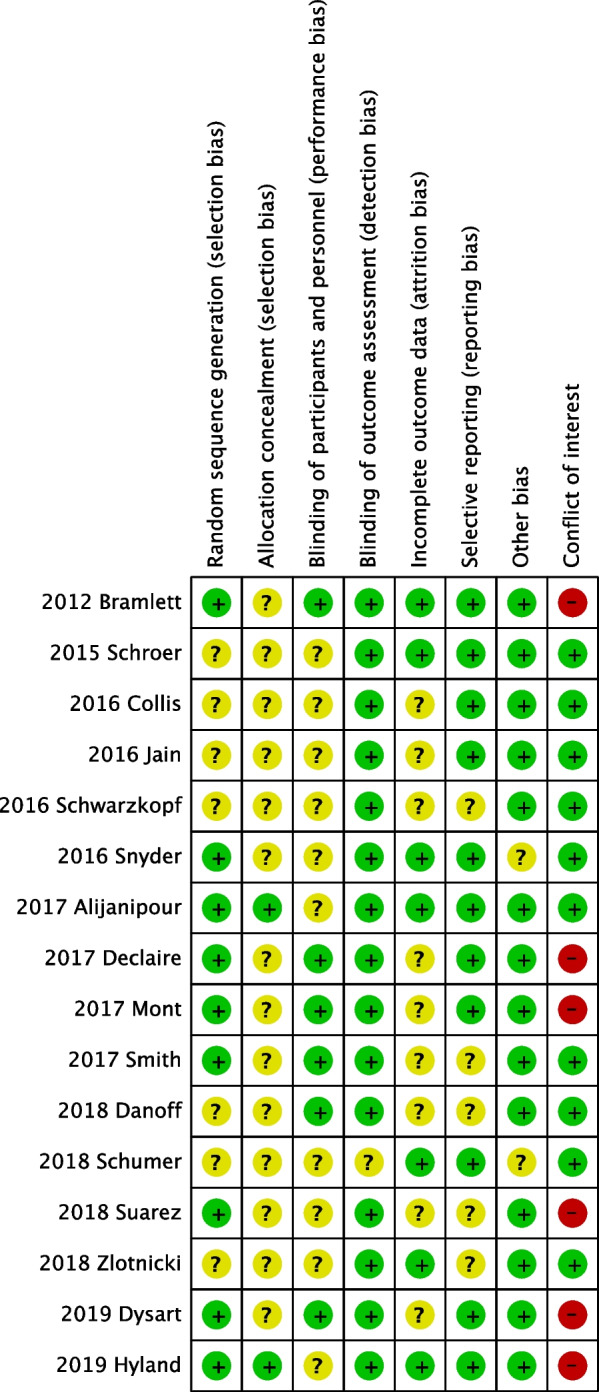
Methodological quality of included studies

**Fig. 3:**
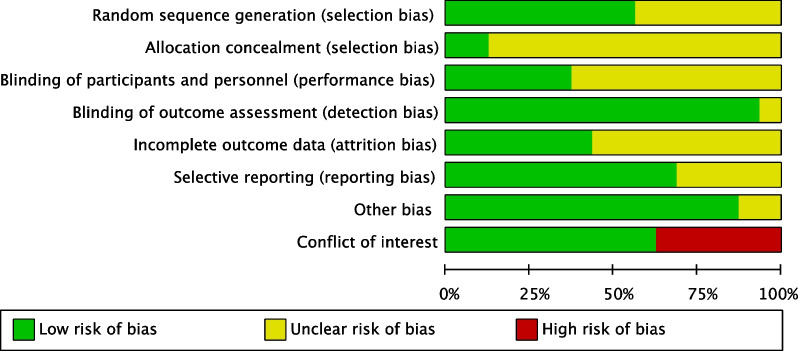
Risk-of-bias assessment of included studies

**Fig. 4:**
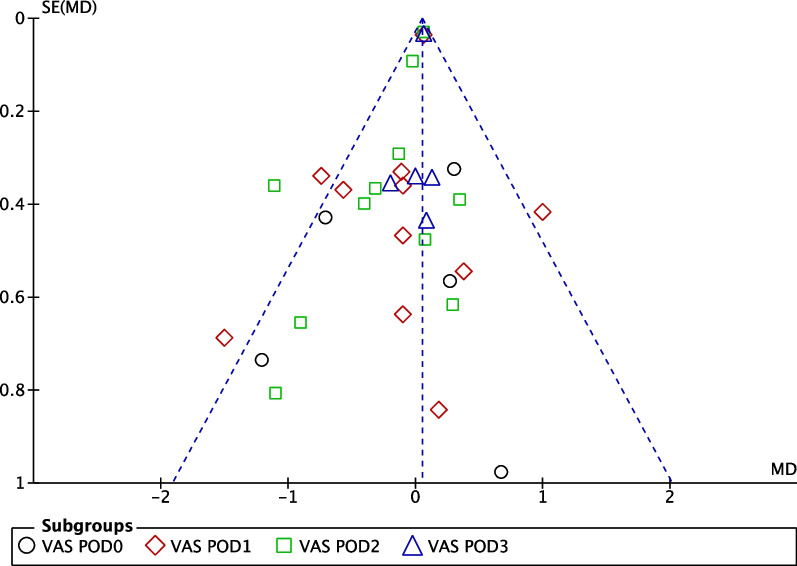
Funnel plot of VAS score. VAS POD1 of Declaire 2017 was not included in the funnel plot due to extremely high standard error (MD = 0.69, SE = 3.08)

The methodological quality of the 16 RCTs was summarized using RevMan software. Figure 2 shows the methodological quality of the included studies, and Fig. 3 displays the evaluation of risk of bias by percentage. Figure 4 depicts the funnel plot of the primary outcome, namely the VAS score. One study, VAS POD1 of Declaire 2017 [30], was not included in the funnel plot due to extremely high standard error (MD = 0.69, SE = 3.08). Asymmetries were noted in postoperative day 0 (POD0), POD1 and POD2, and publication bias cannot be ruled out.

### Primary outcome: postoperative pain score

**Fig. 5:**
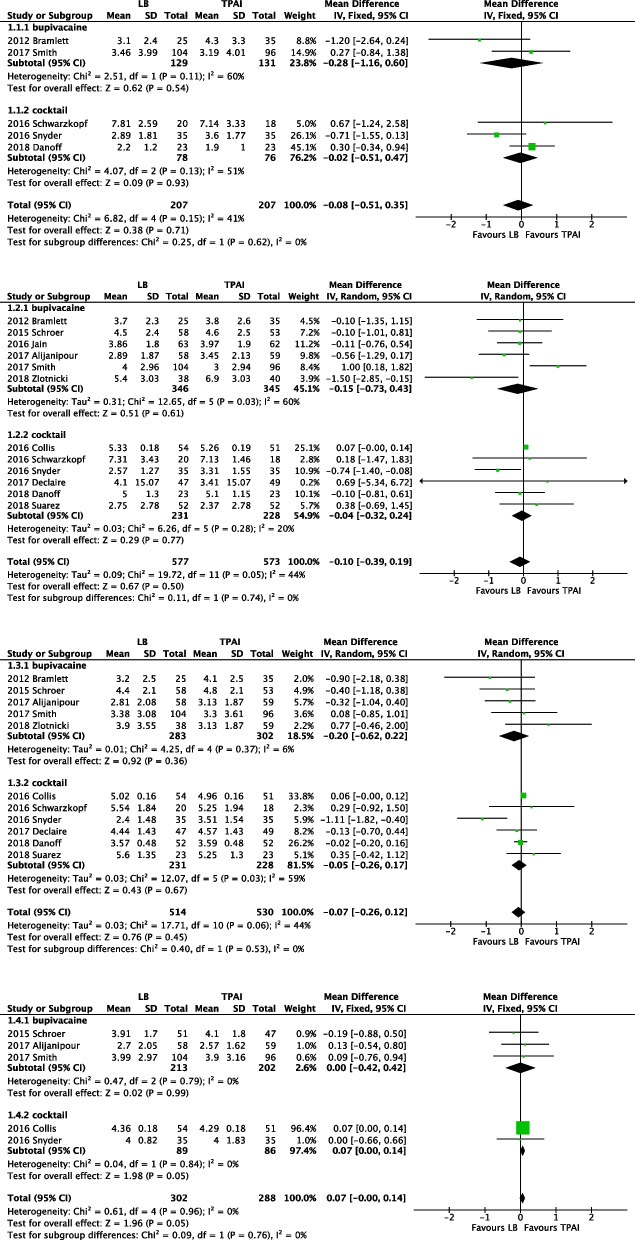
Forest plot of the VAS during postoperative day. From top to bottom are POD 0, POD1, POD2 and POD3

Subgroup analyses of the LB group versus standard bupivacaine group and LB group versus cocktail group were conducted to reduce the possible risk of bias. The assessment time was divided into PODs 0–3 (Fig. 5).

#### POD 0

A meta-analysis of two studies [23, 31] with 260 participants did not reveal a significant difference between the LB and the bupivacaine subgroups (*p* = 0.54).

A meta-analysis of three studies [27, 28, 32] with 154 participants did not reveal a significant difference between the LB and the cocktail subgroups (*p* = 0.93).

#### POD 1

A meta-analysis of six studies [23, 24, 26, 29, 31, 34] with 691 participants did not reveal a significant difference between the LB and the bupivacaine subgroups (*p* = 0.61).

A meta-analysis of six studies [25, 27, 28, 30, 32, 33] with 459 participants did not report a significant difference between the LB and the cocktail subgroups (*p* = 0.77).

#### POD 2

A meta-analysis of fie studies [23, 24, 29, 31, 34] with 566 participants did not show a significant difference between the LB and the bupivacaine subgroups (*p* = 0.36).

A meta-analysis of six studies [25, 27, 28, 30, 32, 33] with 459 participants did not reveal a significant difference between the LB and the cocktail subgroups (*p* = 0.67).

#### POD 3

A meta-analysis of three studies [24, 29, 31] with 415 participants did not reveal a significant difference between the LB and the bupivacaine subgroups (*p* = 0.99).

A meta-analysis of two studies [25, 28] with 175 participants revealed a borderline difference between the LB and the cocktail subgroup (*p* = 0.05). The VAS score was slightly higher in patients who received LB than in patients who received the cocktail injection.

### Secondary outcome

#### Consumption of morphine equivalents

**Fig. 6:**
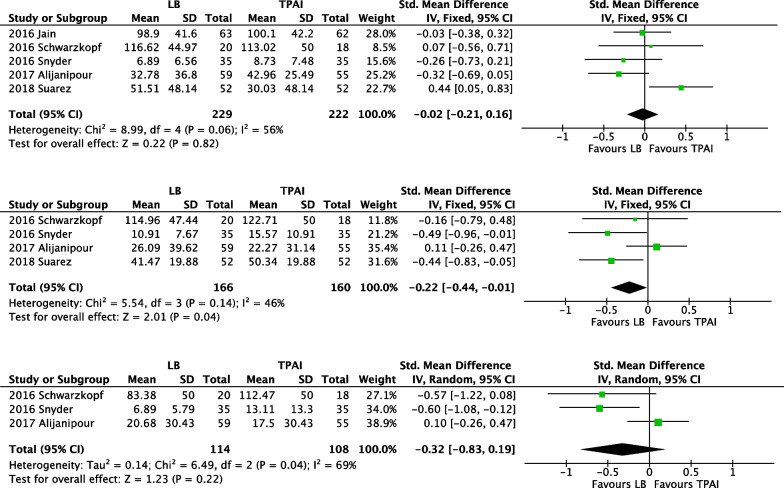
Forest plot of morphine consumption equivalents. From top to bottom are postoperative 0–24 h, 24–48 h and 48–72 h

Morphine consumption was divided into three time periods: postoperative 0–24 h, 24–48 h, and 48–72 h (Fig. 6).

Morphine consumption during 0–24 h.

A meta-analysis of five studies [26–29, 33] with 451 patients revealed no significant difference between the two groups (*p* = 0.82).

Morphine consumption during 24–48 h.

A meta-analysis of four studies [27–29, 33] with 326 patients revealed that morphine consumption in the LB group was less than in the TPAI group (*p* = 0.04).

Morphine consumption during 48–72 h.

A meta-analysis of three studies [27–29] with 222 patients indicated no significant difference, although morphine consumption appeared to be lower in the LB group than in the control group (*p* = 0.22).

#### Incidence of nausea and vomiting

**Fig. 7:**
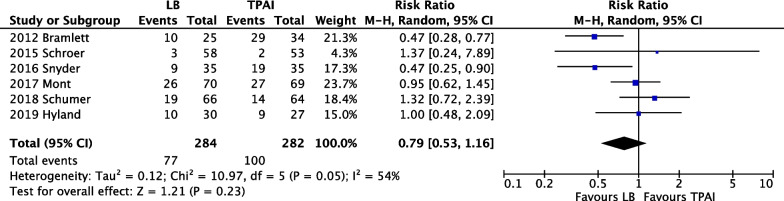
Forest plot of nausea and vomiting incidence

A meta-analysis of six studies [15, 19, 23, 24, 28, 36] with 566 participants revealed no significant difference (*p* = 0.23), although the incidence of nausea and vomiting appeared to be lower in the LB group than in the control group (Fig. 7).

### Length of hospital stay

**Fig. 8:**
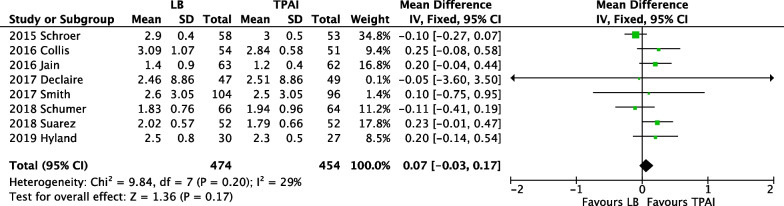
Forest plot of length of hospital stay

A meta-analysis of eight studies [19, 24–26, 30, 31, 33, 36] with 928 participants found that the LB group had a longer hospital stay than the control group, but there was no significant difference between the two groups (*p* = 0.17) (Fig. 8).

### Quality of evidence

The quality of each outcome was evaluated using the GRADE system. Most of the outcome qualities were moderate (Table 2).

**Table 2:** Quality of each outcome of TKA using GRADE system

Outcomes	No. of included studies	No. of patients		(S)MD or RR (95%CI)	Heterogeneity	Quality of evidence (GRADE)
		LB	TPAI			
VAS at POD0 (Bupivacaine)	[23, 31]	129	131	− 0.28 [− 1.16, 0.60]	I2 = 60%, P = 0.11	Moderate^2^
VAS at POD0 (Cocktail)	[27, 28, 32]	78	76	− 0.02 [− 0.51, 0.47]	I2 = 51%, P = 0.13	Low^1,2^
VAS at POD1 (Bupivacaine)	[23, 24, 26, 29, 31, 34]	346	345	− 0.15 [− 0.73, 0.43]	I2 = 60%, P = 0.03	Low^1,5^
VAS at POD1 (Cocktail)	[25, 27, 28, 30, 32, 33]	231	228	− 0.04 [− 0.32, 0.24]	I2 = 20%, P = 0.28	Moderate^1^
VAS at POD2 (Bupivacaine)	[23, 24, 29, 31, 34]	283	302	− 0.20 [− 0.62, 0.22]	I2 = 6%, P = 0.37	Moderate^1^
VAS at POD2 (Cocktail)	[25, 27, 28, 30, 32, 33]	231	228	− 0.05 [− 0.26, 0.17]	I2 = 59%, P = 0.03	Low^1,5^
VAS at POD3 (Bupivacaine)	[24, 29, 31]	213	202	0.00 [− 0.42, 0.42]	I2 = 0%, P = 0.79	High
VAS at POD3 (Cocktail)	[25, 28]	89	86	0.07 [0.00, 0.14]	I2 = 0%, P = 0.84	Moderate^1^
Opioid consumption at 24 h	[26–29, 33]	229	222	− 0.02 [− 0.21, 0.16]	I2 = 56%, P = 0.06	Moderate^1^
Opioid consumption at 48 h	[27–29, 33]	166	160	− 0.22 [− 0.44, − 0.01]	I2 = 46%, P = 0.14	Moderate^1^
Opioid consumption at 72 h	[27–29]	114	108	− 0.32 [− 0.83, 0.19]	I2 = 69%, P = 0.04	Moderate^1^
Nausea and vomiting	[15, 19, 23, 24, 28, 36]	284	282	0.79 [0.53, 1.16]	I2 = 54%, P = 0.05	High
Length of hospital stay	[19, 24–26, 30, 31, 33, 36]	474	454	0.07 [− 0.03, 0.17]	I2 = 29%, P = 0.20	Moderate^1^

GRADE Working Group grades of evidence

High quality: Further research is very unlikely to change our confidence in the estimate of effect

Moderate quality: Further research is likely to have an important impact on our confidence in the estimate of effect and may change the estimate

Low quality: Further research is very likely to have an important impact on our confidence in the estimate of effect and is likely to change the estimate

Very low quality: We are very uncertain about the estimate

^1^Risk of bias

^2^Inconsistency

^3^Indirectness

^4^Imprecision

^5^Publication bias

## Discussion

The findings of this meta-analysis provided moderate-quality evidence that the LB group had lower morphine consumption equivalent in the second 24 h than the TPAI group. However, the overall postoperative pain scores, morphine consumption equivalents in the first and the third 24 h, the incidence rate of nausea and vomiting, and the length of hospital stay did not differ significantly between the LB and TPAI groups for patients who underwent TKA.

The liposomal bupivacaine was hardly enough to completely blind the surgeon who administered the drugs because it has a cloudy liquid appearance and is more viscous than the conventional pain cocktail, which also contains bupivacaine or ropivacaine [30]. Smith et al. consequently instructed the surgeon to leave the operation room while trained medical assistants administered the drugs [31]. Snyder et al. transferred the LB to a sterile syringe covered in an opaque bandage [28]. Most studies [19, 24–29, 33, 34, 36] simply excluded the surgeon from any outcome assessment or data analysis; however, this approach could not eliminate the performance bias.

The dose range for LB was 106–532 mg. Hu et al.’s [37] comparison of various doses of LB revealed a quantitative similarity in the plasma concentration versus time profiles as well as a lower incidence of adverse events in the group of LB ≤ 226 mg than in the group of LB > 226 mg. Most of the RCTs used LB in a dose of 266 mg, which is the maximum FDA-approved dose, in a single 20 mL vial. Its widespread use may be attributed to a sufficient plasma concentration and fewer adverse events.

The pharmacokinetics of LB were also demonstrated by Hu et al. [37], with the first peak occurring within an hour after injection and the second peak 12–36 h later. The present study found a similar VAS score and morphine consumption between the LB and TPAI groups in POD 0 and POD 1. The morphine rescue dose was lower in the LB group up until POD 2, but the incidence of nausea and vomiting was not significantly lower. There was no significant difference in VAS score and morphine consumption between the two groups after POD 3. Overall, LB did not significantly improve the VAS score when compared to TPAI.

A total of six studies [15, 19, 23, 24, 28, 36] evaluated the incidence of nausea and vomiting. There was no significant difference in the incidence of nausea and vomiting, although the consumption of morphine equivalents in the LB group was less than in the TPAI group during the postoperative 24–48-h period. This may be attributed to no significant difference in morphine consumption and an insufficient number of included studies.

A total of eight studies [19, 24–26, 30, 31, 33, 36] reported the length of hospital stay. Several factors influence the duration of hospital stay after TKA, including age, sex, and preoperative hemoglobin. Postoperative pain and functional recovery are also critical factors. The lack of a significant difference in VAS score may implicate that the processes of rehabilitation and functional recovery were similar for both groups, regardless of LB or TPAI usage. There is currently no conclusive evidence that LB can shorten the length of hospital stay.

Several RCTs compared the cost of LB with that of standard periarticular injection. Collis et al. [25] reported that the total cost of LB injection was $285 US, which was more than seven times the cost of the modified Ranawat suspension (Ropivacaine, epinephrine, ketorolac, clonidine) ($40 US). Hyland et al. [19] reported that the cost of LB, which was approximately $300.66 US per patient in 2019, was more than 17 times that of the PAI (approximately $16.83 US). The nonsignificant outcome differences found in this study seems not support for the use of LB. However, there may be variations in the cost of LB in different countries/regions. We consider that LB may become more competitive if its cost is cheap enough.

The pain-relieving effect of periarticular multimodal drug injection (PMDI) has been widely reported [7–12]. However, it is challenging to interpret the results of studies that combined local anesthetics with epinephrine, nonsteroidal anti-inflammatory drugs (NSAIDs), and morphine. Some studies used standard bupivacaine injection as the control group, whereas others used ropivacaine or cocktail agents. Despite this, the present study divided the population into two groups on the basis of whether standard bupivacaine or a cocktail was used as the control. The components in either the bupivacaine or cocktail subgroup remain inconsistent. For instance, some studies combined epinephrine, whereas others did not. The risk of bias could not be eliminated even after the subgroups were classified. Despite the possible bias, the current clinical situation was more consistent with the different PAI components. The most optimal components are still inconclusive. There are increasing studies exploring the specific effect of single agents in the PMDI. A comparison with LB will provide more valid results if the most acceptable agents are confirmed.

### Strengths and limitations

Only RCTs were included in the present meta-analysis, and of similar studies, to the best of our knowledge, this study analyzed the most RCTs. Most of the RCTs explained their randomization method, and all of them blinded the participants and outcomes assessors. The present study not only followed the PRISMA guidelines but also used the GRADE system to evaluate the evidence level of each outcome. More and newer data were included than in previous meta-analyses to obtain more compelling results.

There are several limitations in the present study. First, there were multiple TPAI components, and differences of the components were observed in most of the RCTs, contributing to the risk of bias when comparing them to each other. Second, different RCTs used different time units. For example, some studies used POD 1, POD 2, and POD 3. In some other studies, the time units were the first 24 h, second 24 h, at 24 h, or at 48 h. There might be some bias introduced by combining these different time units. Third, this study contains only a small number of RCTs. Although 16 RCTs were identified, not every study provided the outcomes expected. For instance, only five RCTs provided the VAS score on the operation day. Additionally, because the majority of RCTs had fewer than 50 participants, it was challenging to determine the incidence rate of nausea and vomiting, which may be better observed in a large study population. Fourth, various anesthesia methods were adopted in these RCTs, with spinal anesthesia being the most common choice. However, spinal anesthesia may also have an effect on postoperative pain, and this issue was not addressed in our study. Finally, all of the RCTs were conducted in the USA. Therefore, it is difficult to generalize the results to other countries or races.

### Implications for practice and research

Analyses of functional recovery, range of motion of the joints, or other complications besides nausea and vomiting were not performed due to the limited availability of data. These are valuable outcomes in addition to the VAS score. Future studies may consider analyzing more postoperative parameters.

## Conclusion

Morphine consumption equivalents were lower in the LB group in postoperative 24–48 h. LB administration during TKA is not superior to TPAI in terms of postoperative VAS, nausea and vomiting incidence, and length of hospital stay. Studies with larger sample size are needed to validate our findings.

## Data Availability

All data generated or analyzed during this study are included in published articles.
